# Interactions between Severe Allergy and Anxiety in Anti-SARS-CoV-2 Vaccinees

**DOI:** 10.3390/vaccines10122047

**Published:** 2022-11-30

**Authors:** Chiara Asperti, Giovanni Benanti, Giuseppe A. Ramirez, Marco Russo, Benedetta Vai, Barbara Bramé, Naomi Viapiana, Serena Nannipieri, Maria Bernadette Cilona, Martina Mazzetti, Simone Zuffada, Valentina Elisabetta Di Mattei, Francesco Benedetti, Lorenzo Dagna, Mona-Rita Yacoub

**Affiliations:** 1 Unit of Immunology Rheumatology, Allergy and Rare Diseases, IRCCS Ospedale San Raffaele, 20132 Milan, Italy; 2 Faculty of Medicine, Università Vita-Salute San Raffaele, 20132 Milan, Italy; 3 Psychiatry and Clinical Psychobiology, Division of Neuroscience, IRCCS San Raffaele Scientific Institute, 20132 Milan, Italy; 4 Nuovo Ospedale di Legnano, Uoc Medicina Generale, 20025 Legnano, Italy; 5 Clinical and Health Psychology Unit, IRCCS San Raffaele Scientific Institute, 20132 Milan, Italy

**Keywords:** vaccine, COVID-19, SARS-CoV-2, allergy, anxiety

## Abstract

Severe drug allergy affects patient hesitancy to new treatments, posing unprecedented challenges to anti-SARS-CoV-2 vaccination campaigns. We aimed to analyze the psychological profile of vaccinees with a history of severe allergy in comparison to subjects with a milder allergy history. Patients attending a dedicated vaccination setting were administered an anonymized questionnaire including clinical data and the State-Trait Anxiety Inventory (STAI) scale (score range 20–80). Patients were also asked whether being in a protected setting affected their attitude toward vaccination. Data are expressed as median (interquartile range). We enrolled 116 patients (78% women), of whom 79% had a history of drug anaphylaxis. The median state anxiety score was 36.5 (30–47.2), while the trait anxiety score was 37 (32–48). State anxiety was higher in those with severe than mild allergy [39 (32–50) vs. 30 (25–37); *p* < 0.001], with the highest score found in a patient with previous drug anaphylaxis (42.5 [32–51.7]). More than 50% of patients reported that being in a protected setting had lowered their anxiety. Severe allergy is associated with a higher burden of situational anxiety in the setting of vaccination without affecting patient constitutional (trait) levels of anxiety. Vaccination in dedicated facilities might overcome issues related to hesitancy and improve patients’ quality of life.

## 1. Introduction

Anaphylaxis and severe allergic reactions constitute life-threatening events occurring with an estimated incidence of 4–5 cases per 100,000 persons per year [1]. Severe allergy survivors also face long-term psychological sequelae affecting their quality of life [1,2,3]. These subjects often develop a generalized sense of insecurity and anxiety, and they tend to be wary about changes in their medication and the administration of new drugs [3].

This might pose a relevant challenge in the setting of the ongoing severe acute respiratory syndrome coronavirus 2 (SARS-CoV-2) pandemic, which has prompted mass exposure to a vast array of newly developed treatments and vaccines [4].

True systemic hypersensitivity against vaccine is rare. For the Pfizer/BioNTech BNT162B2 COVID-19 vaccine, it occurs in 11.1 cases per 1 million doses [5]. Furthermore, 80% of patients with hypersensitivity reactions (HRs) to vaccines had a history of positive allergic reactions to food, drugs, or insect sting [6]. According to the European Academy of Allergy and Clinical Immunology (EAACI), only patients with an established allergy to vaccine components have an absolute contraindication to vaccination [7]. Still, a relevant number of people have been inappropriately considered at-risk, and allergists have been insofar fundamental in assessing and identifying actual at-risk subjects [8]. As an example, in Hong Kong, allergist-led vaccination sessions had a vaccine recommendation rate of 98.9%, compared to 81% in the non-allergist-led one [9].

Nevertheless, people with a history of severe allergy should be vaccinated by staff able to recognize and treat allergic reactions [5]. In Italy, national guidelines recommend vaccination in a “protected setting” consisting in a medical center with dedicated staff and prolonged surveillance for these patients in order to protect their safety. Patients can receive indication directly from their allergist or can be referred by standard vaccination centers if deemed necessary, usually having a history of previous anaphylaxis, multiple drug reaction, previous suspected hypersensitivity reaction to COVID-19 vaccines (but negative skin tests to excipients), severe or uncontrolled asthma or chronic spontaneous urticaria (CSU) [10,11,12].

However, in the context of these “protected settings”, allergists could also have a role in overcoming vaccine hesitancy by this special population [13].

In this study, we aimed to investigate anxiety levels of patients with a history of severe HR undergoing vaccination in “protected settings”: we have compared state and trait anxiety levels between a Severe Allergic Group (SAG) and a group constituted by subjects with mild allergic background (Mild Allergic group = MAG). We also evaluated the potential effects of vaccination-protected setting on psychological well-being.

## 2. Materials and Methods

### 2.1. Patients and Procedures

We studied a cohort of consecutive allergic patients being referred to dedicated vaccination facilities at IRCCS San Raffaele Hospital (Milan, Italy) and Legnano Hospital (Legnano, Milan, Italy) from 8 October 2021 to 13 April 2022. Patients referred to these facilities had been deemed at risk for vaccine-related HRs [10] by either their allergist or a standard vaccination center. Enhanced safety measures included trained personnel for prompt resuscitation and prolonged post-vaccine observation for one hour. According to the provisions of our Hospital Institution, all subjects received the BNT162b2 (Comirnaty^®^) vaccine.

Based on clinical history, patients were subdivided first into SAG or MAG groups. SAG patients had a history of anaphylaxis or severe HR to drugs, foods, or insect stings (defined as grade two or higher of the word allergy organization classification [14]). MAG included patients with no severe allergic history (non-severe food allergy, well-controlled asthma or CSU, rhinitis/conjunctivitis, atopic dermatitis, and allergic contact dermatitis).

Next, we performed a second analysis by dividing our population into a Severe Drug Reaction (SDR) group and a non-SDR group in order to ascertain the specific role of drug allergy history.

Data collection was performed in the post-vaccine observation timeframe through an anonymous questionnaire. The questionnaire was designed in compliance with the European Commission guidelines for anonymization in such a way that patient identification was impossible for the investigators or other subjects [15]. For these reasons, the study did not require formal approval by the local Institutional Review Board. Collected data included patient demographics (gender, age range), general clinical history (comorbidities, ongoing therapies), and allergic history (previous severe reaction to vaccines or drugs, foods, insect stings, and respiratory or contact allergy). The number and type of previous anti-SARS-CoV-2 vaccines were also recorded. Anxiety was measured through the STAI-Y questionnaire. STAI-Y is a validated questionnaire, initially devised in 1970 and later revised in 1983 by Spielberger, one of the most used tools to analyze anxiety in medical research [16]. It provides a quantitative measurement of anxiety, separately analyzing the habitual proneness to anxiety (trait anxiety) and the in-the-moment anxiety to a specific event (state anxiety) [16]. Each section comprises 20 items, presented in both positive and negative forms, graded 1 to 4, with a total score ranging from 20 to 80. Higher scores are positively correlated with higher levels of anxiety; a cut-off score >39, as suggested by the literature, has been used to define clinically significant anxiety symptoms [17]. Patients were also asked whether having been referred to a dedicated facility had made them feel more or less anxious about vaccination.

### 2.2. Statistical Analysis

Shapiro-Wilk normality tests were performed to assess whether continuous variables had or not a normal distribution. Due to the non-normal distribution of continuous variables, non-parametric tests were employed. Correlation between continuous variables was performed with Spearman’s test. Mann–Whitney’s U-test was used to compare continuous variable trends between groups. The distribution of categorical variables among groups was compared using the Chi-square test with Fisher’s exact correction. Continuous variables are expressed as median (interquartile range, IQR) unless otherwise specified. Categorical variables are reported as absolute numbers (percentages). RStudio 4.2.1. and JASP 0.16.0.0 were used for statistical analysis.

## 3. Results

SAG and MAG encompassed 89 and 27 subjects, respectively. In the SAG, 86% were women, and the most represented age range was 55–59 years. 78% of them reported previous drug anaphylaxis, and 56% food anaphylaxis. Allergic comorbidities (rhinitis, atopic dermatitis, asthma, pollen-fruit syndrome) were present in 67% of SAG and 56% of MAG. However, except for a history of food allergy, no significant differences were detected between SAG and MAG regarding the prevalence of allergic comorbidities. The demographic and clinical features of patients are shown in Table 1. SDR e non-SDR encompassed 69 and 47 patients, respectively. Demographic and clinical features of patients are shown in Table S1). Symptoms during post-vaccine observation were reported by 12.9%, but only 4.3% were suggestive of HR. In detail, four patients reported local pain in the injection site, four patients reported skin rash, one diffuse pruritus, one “oral itching”, one headache, one heartburn, and one had a hypertensive episode. None of them reported systemic HRs or other any other severe adverse effect.

Regarding the psychological impact of vaccine administration in a “protected setting”, 60.3% answered that it made them feel less anxious, while only 9.4% were more anxious due to the hospital setting. Among the subgroup of patients with previous drug anaphylaxis, a significantly higher number of patients (71% in SDR vs. 50% in non-SDR, *p* = 0.015) reported that being in a “protected setting” made them feel less anxious (Figure S1).

**Figure 1:**
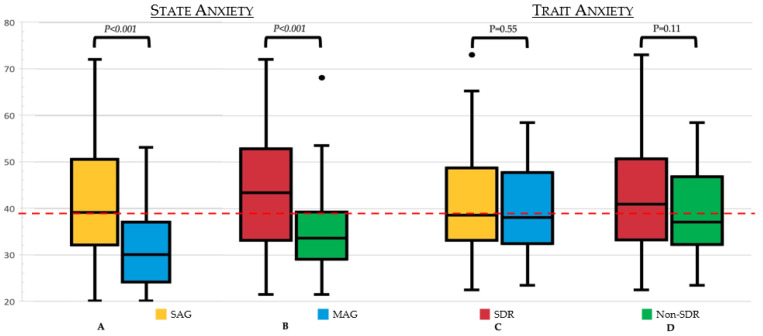
(**A**) State anxiety levels according to the STAY-Y tool in SAG and MAG. (**B**) State anxiety levels according to the STAY-Y tool in SDR and non-SDR groups. (**C**) Trait anxiety levels according to the STAY-Y tool in SAG and MAG. (**D**) Trait anxiety levels according to the STAY-Y tool in SDR and non-SDR groups. *p* values are shown on each of the graphs. The dotted line represents the clinical cut-off to define relevant anxiety level (>39). Using the STAY-Y tool, we assessed the anxiety level of subjects undergoing COVID-19 vaccination. First of all, we observed that patients with severe allergy background have a higher anxiety level during vaccination (39 IQR [32–50] vs. 30 IQR [24.5–36.4]), and the difference was even more evident in subjects with previous severe drug allergy (42.5 IQR [32–51.7] vs. 32.5 IQR [28–37.7]). On the contrary, no difference was observed in their trait anxiety (usual properness toward anxiety) between SAG and MAG (37.5 IQR [32.0–48.0] vs. 37 IQR [31.7–47) or between the SDR group and the non-SDR group (40 IQR [32–49] vs. 36 IQR [31.5–46]). Of note, 39 is usually used as the cut-off to define a relevant state or trait anxiety, so it appears that MAG and non-SDR groups in the median did not have a relevant anxious state during vaccination, while both SAG and SDR groups presented with clinically relevant anxiety. SAG = severe allergy group; MAG = mild allergy group; SDR = severe drug reaction.

Regarding the assessment of anxiety with the STAI-Y questionnaire, we found a statistically significant correlation between state anxiety and trait anxiety (rho = 0.580, *p* = 0.001). Gender did not correlate with a difference in anxiety level, while age range had a negative correlation with both state *p* = 0.033, rho = −0.200) and trait anxiety (*p* = 0.030, rho = −0.200), meaning that younger patients were more anxious than older patients both in general and during vaccination (Figures S2 and S3). SAG subjects had significantly greater post-vaccination state anxiety than subjects who reported no severe reactions in their history (*p* < 0.001). This trend was replicated in the SDR group (*p* < 0.001), where the difference between median state anxiety SDR and non-SDR (42.5 IQR [32–51.7] vs. 32.5 IQR [28–37.7]) was even greater than between SAG and MAG (39 [IQR 32–50] vs. 30 [IQR 24.5–36.5]). Moreover, both SAG and SDR groups had a median state anxiety level that was clinically significant. However, no significant differences in trait anxiety either between SAG and MAG or between SDR and the non-SDR group were found (Figure 1). Of note, subdividing patients according to the number of previous COVID-19 vaccinations yielded no significant differences in state or trait anxiety levels.

Other clinical features, such as atopy, CSU, and non-allergic comorbidities, were not associated with different levels of anxiety. Anxiety did not correlate with the onset of post-vaccination symptoms either in SAG or in MAG.

## 4. Discussion

Widespread vaccination against COVID-19 represents the current goal of public health. Allergists have been central insofar in order to define the minority of patients with contraindications to vaccination. Furthermore, as stated in the EAACI position paper, allergists should reassure patients with a severe allergic background in order to increase their compliance toward vaccines [5]. The so-called “infodemic”, i.e., uncontrolled spreading of inflated news and fake news regarding COVID-19, has heightened anxiety concerning vaccine safety [18,19]. Anxiety has, in turn, long been regarded as an important issue in the allergic population due to the known long-lasting harmful effects on psychological balance observed in anaphylaxis survivors a [3].

Yet, the link between anxiety and allergy and their clinical consequences is complex and not entirely explored. Several researchers reported a pathophysiological link between anxiety and the onset of nocebo reactions. Higher anxiety states correlate whit nocebo during drug provocation tests [20,21]. Nocebos, in turn, can affect 78% of adverse reactions to anti-COVID-19 vaccines, according to a recent meta-analysis of randomized controlled trials [22].

Our study aimed to explore in detail the relationship between anxiety and allergy in the context of COVID-19 vaccination. Our data confirmed the correlation between a history of a severe allergic reaction, especially, to drugs, and anxiety. Specifically, we found that patients with severe allergies had a higher state anxiety score compared to patients with mild allergies. We also observed a non-significant trend towards higher levels of trait anxiety in patients with a severe allergy. The association between severe allergy and state anxiety was particularly strong in SDR patients.

On the other hand, the majority of subjects (especially those with a severe allergic background) felt reassured by an allergist-led protected setting. This highlights the importance of considering the psychological profile of allergic vaccinees and supports the role of allergists in overcoming vaccine hesitancy.

To our knowledge, the relationship between anxiety and allergy has not previously been evaluated in the context of COVID-19 vaccination. Nonetheless, our study does have limitations. First, our population was only constituted by allergic patients (both mild and severe) since, by definition, only this population was directed to our protected vaccinal sessions. Still, our aim was to describe the impact of anxiety in severely allergic patients, and therefore MAG represented a reasonable study comparator. In fact, significant differences were observed between these groups. Second, clinical and demographic data were self-reported and anonymized, preventing post hoc validation of acquired data or extension beyond predefined analyses. However, patient referral to vaccination in a protected setting was based on a physician review of individual clinical data. Third, while the STAY questionnaire is a validated and widely used tool in medical research, it does not cover the whole spectrum of anxiety and could underestimate the impact of some confounding factors. Moreover, we did not collect data about patient education level and knowledge or beliefs about vaccines or drugs. However, we detected no significant difference in state anxiety by vaccine dose number (i.e., primary cycle or booster doses). This could imply that even vaccine experience does not affect vaccine-related anxiety. Fourth, the patient sample size was relatively small, and there was no long-term follow-up, limiting the detection of infrequent or delayed events and potential correlation with anxiety.

## 5. Conclusions

In summary, we showed how patients with a severe allergic background, especially severe drug allergy, have a significant psychological burden and concern about new vaccines. A protected setting led by an allergist not only could be effective in ensuring vaccination safety in patients with clinically relevant allergic history [8] but could also increase patient compliance toward vaccinations.

## Figures and Tables

**Table 1:** Demographic and clinical features. Abbreviations: (HR) = Hypersensitivity reactions; (AD) = atopic dermatitis; (ACD) = allergic contact dermatitis; (CSU) = chronic spontaneous urticaria; (SPT) = Skin Prick Test; AntiH1 = antiH1 antihistamine; (STAI) = State-Trait Anxiety Inventory; (IQR) = Interquartile range, (NA) = not applicable.

	Total Sample	Severe Allergic Group	Mild Allergic Group	*p*
N (100%)	116 (100)	89 (77)	27 (23)	NA
Females: n (%)	90 (78)	76 (86)	15 (51)	<0.010
Age Class: median (IQR)	47 (37–57)	47 (37–57)	47 (32–57)	ns
Drug anaphylaxis: n (%)	70 (60)	70 (79)	0 (0)	<0.001
Drug HRs ≥ 2: n (%)	49 (42)	49 (56)	0 (0)	<0.001
Food anaphylaxis: n (%)	43 (37)	42 (48)	0 (0)	<0.001
Allergic comorbidities: n (%)	75 (66)	59 (67)	15 (56)	ns
Rhinitis/conjunctivitis: n (%)	37 (32)	30 (34)	7 (26)	ns
Asthma: n (%)	33 (28)	26 (30)	7 (26)	ns
AD: n (%)	33 (28)	27 (31)	5 (22)	ns
ACD: n (%)	29 (25)	27 (30)	2 (7)	<0.050
CSU: n (%)	12 (10)	9 (10)	3 (11)	ns
Food allergy: n (%)	47 (41)	42 (48)	5 (22)	<0.050
Hymenoptera allergy: n (%)	18 (16)	19 (22)	3 (11)	ns
Positive SPT: n (%)	60 (52)	51 (57)	9 (33)	<0.050
AntiH1 therapy: n (%)	49 (42)	39 (44)	10 (37)	ns
Inhaled asthma therapy	25 (21)	19 (21)	6 (22)	ns
Comorbidities: n (%)	19 (16)	16 (18)	3 (11)	ns
Dose 1: n (%)	16 (14)	12 (14)	4 (15)	ns
Dose 2: n (%)	46 (40)	36 (41)	9 (33)	ns
Dose 3: n (%)	52 (45)	38 (43)	14 (52)	ns
Symptoms: n (%)	15 (13)	13 (15)	2 (7)	Ns
STAI-state: median (IQR)	36.5 (30.0–47.2)	39.0 (32.0–50.2)	30. 0 (24.5–36.5)	<0.050
STAI-trait: median (IQR)	37.0 (32.0–48.0)	37.50 (32.0–48.0)	37.0 (31.5–47.0)	Ns

## Data Availability

The data presented in this study are available on request from the corresponding author. The data are not publicly available due to privacy.

## Supplementary Materials

The following supporting information can be downloaded at: https://www.mdpi.com/article/10.3390/vaccines10122047/s1, Figure S1: Answers to “Do you think that protected setting made you feel More Anxious, Less anxious or had no impact on your anxiety?” in the different subgroups; Figure S2: correlation between age range and state anxiety; Figure S3: correlation between age range and trait anxiety. Table S1: Demographic and clinical features.
