# Supplementary material for: Interactions between Severe Allergy and Anxiety in Anti-SARS-CoV-2 Vaccinees
Source: Vaccines (Basel). 2022 Nov 30;10(12):2047. doi: 10.3390/vaccines10122047 (PMC9783305; doi:10.3390/vaccines10122047)
Supplement: Supplementary file 1 [file vaccines-10-02047-s001.zip › vaccines-2030421-supplementary.pdf]

vaccines

MDPI

Supplementary Figures

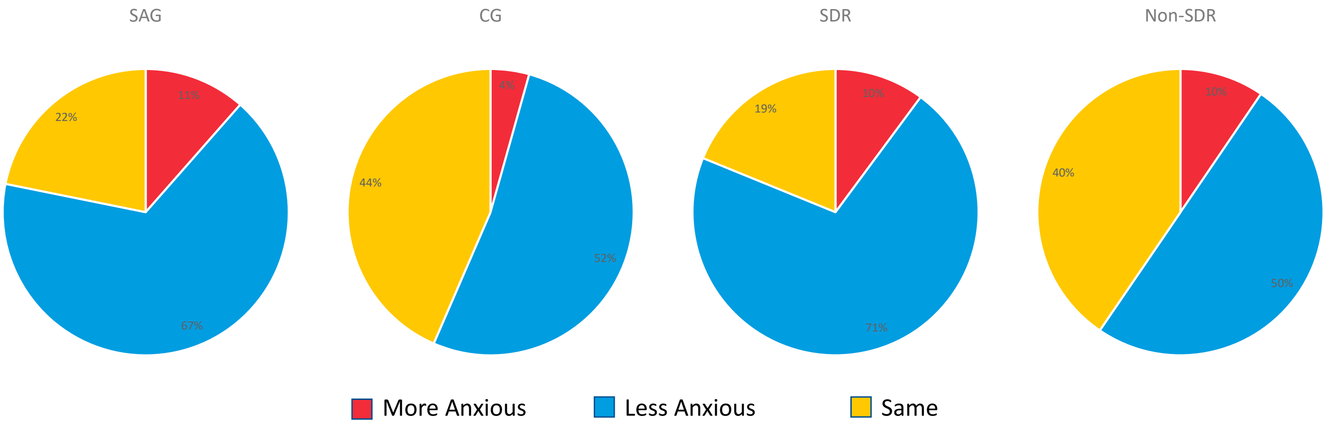

Figure S1 "Do you think that protected setting made you feel More Anxious, Less anxious or had no impact on your anxiety?" – Here are shown how patients in the different group and subgroups answered to the question about the psychological impact of the allergist-led protected setting

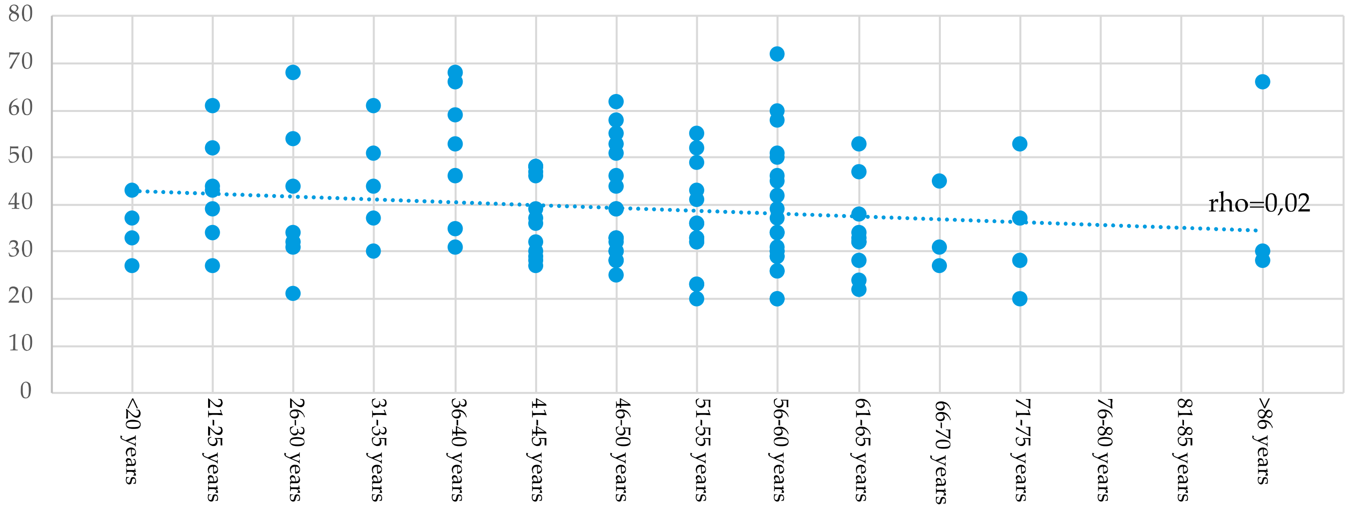

Figure S2 Age Range - State Anxiety correlation: age range had a negative correlation with state anxiety, i.e. younger subject had a tendency towards an higher state anxiety

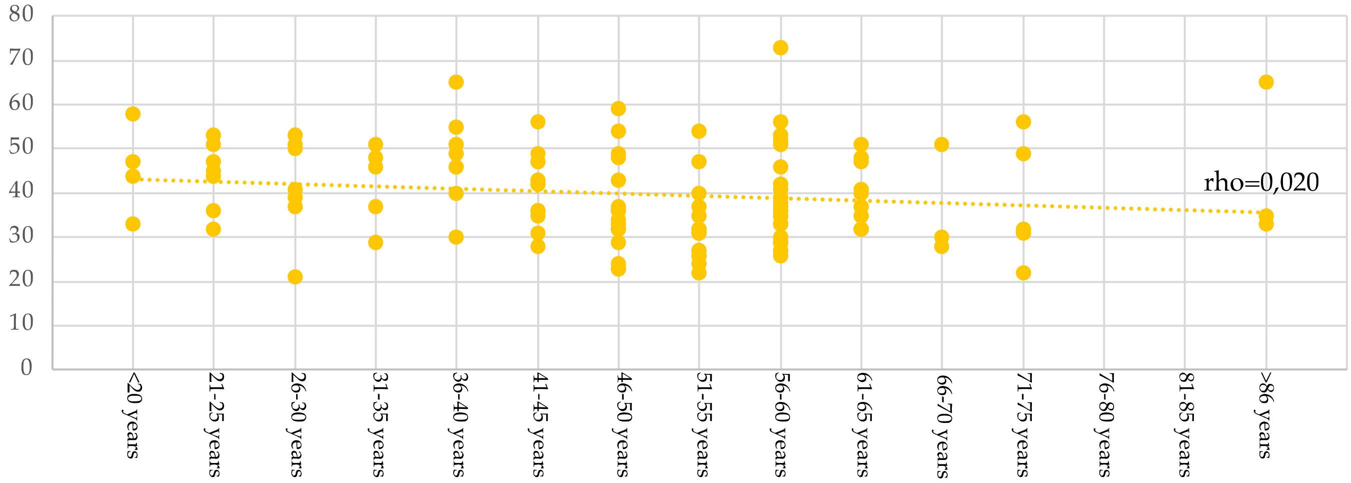

Figure S3 Age Range - Trait Anxiety correlation: age range had a negative correlation with trait anxiety, i.e. younger subject had a tendency towards an higher trait anxiety

Supplementary table

	Total Sample n (%)	SDR Group	Non-SDR Group	P value
n	116 (100)	70 (60)	46 (40)	
Females: n (%)	90 (78)	58 (83)	28 (60)	ns
Age: median (IQR)	47 (37-57)	47 (32-57)	47 (37-57)	ns
Drug anaphylaxis: n (%)	70 (60)	70 (79)	0 (0)	p<0.001
Drug HRs ≥ 2: n (%)	49 (42)	39 (56)	9 (20)	p<0.001
Food anaphylaxis: n (%)	43 (37)	28 (40)	15 (33)	ns
Allergic comorbidities: n (%)	75 (66)	47 (67)	28 (61)	ns
Rhinitis/conjunctivitis: n (%)	37 (32)	23 (33)	14 (30)	ns
Asthma: n (%)	33 (28)	18 (26)	15 (33)	ns
AD: n (%)	33 (28)	23 (33)	10 (22)	ns
ACD: n (%)	29 (25)	21 (30)	8(17)	ns
CSU: n (%)	12 (10)	6 (9)	6 (13)	ns
Food allergy: n (%)	47 (41)	29 (41)	18 (3)	ns
Hymenoptera allergy: n (%)	18 (16)	11 (16)	7 (15)	ns
Positive SPT : n (%)	60 (52)	42 (60)	18 (39)	p<0.05
AntiH1 therapy : n (%)	49 (42)	33 (47)	22 (48)	ns
Inhalatory asthma therapy	25 (21)	11 (16)	14 (30)	ns
Comorbidities: n (%)	19 (16)	11 (16)	8 (17)	ns
Dose 1: n (%)	16 (14)	8 (11)	4 (15)	ns
Dose 2: n (%)	46 (40)	29 (41)	17 (37)	ns
Dose 3: n (%)	52 (45)	31 (44)	21 (46)	ns
Symptoms: n (%)	15 (13)	12 (17)	3 (6)	ns
STAI-state: median (IQR)	37.5 (32-48)	40 (32-49)	36 (31.5-46)	p< 0.001
STAI-trait: median (IQR)	37.0 (32.0-48.0)	42.5 (32.0-51.7)	37 (31.7-47)	ns

Supplementary Table 1. Demographic and clinical features. Abbreviations: (HR)= Hypersensitivity reactions; (AD)= atopic dermatitis; (ACD)= allergic contact dermatitis; (CSU)= chronic spontaneous urticaria; (SPT)= Skin Prick Test; AntiH1= antiH1 antihistamine; (STAI)= State -Trait Anxiety Inventory; (IQR)= Interquartile range.

SDR= Severe Drug Reactions
